# Gallbladder agenesis and choledochogastric fistula in patient with history of cholangitis: A case report

**DOI:** 10.22088/cjim.14.4.751

**Published:** 2023

**Authors:** Nasser Malekpour Alamdari, Adel Zeinalpour, Barmak Ghollizadeh, Maryam Abbasi, Faezeh Shams, Hamed Ebrahimi bagha

**Affiliations:** 1 Department of General Surgery, Shahid Modarres Hospital, Shahid Beheshti University of medical sciences, Tehran, Iran

**Keywords:** Cholangitis, Gallbladder Agenesis, Fistula, Gastro-intestinal

## Abstract

**Background:**

Gallbladder agenesis is a biliary tract related congenital malformation with an incidence of 10–65 per 100,000 and associated with other congenital abnormalities. GA is usually asymptomatic, but sometimes patients become symptomatic. The most usual symptoms are jaundice, abdominal pain in the right upper quadrant, nausea and vomiting. We reported a case of GA and choledochogastric fistula in a patient with history of cholangitis.

**Case Presentation:**

A 70-year-old man presented to Emergency Department of Modarres Hospital with jaundice, fever, right upper quadrant abdominal pain, nausea and vomiting. Clinical examination and lab test demonstrated cholangitis. He underwent ultrasonography, abdominopelvic CT scan and ERCP. ERCP revealed a CBD fistula. Due to recurrent symptoms, he underwent operation and hepaticojejunostomy was done.

**Conclusion:**

In our knowledge, the case of GA and choledochogastric fistula is rare. Furthermore, this type of abnormalities rarely presented with cholangitis, so probable anatomical malformation of the biliary tract should always be considered as a differential diagnosis in patients with biliary disease signs and symptoms.

Gallbladder agenesis (GA) is a scarce inborn malformation of biliary tract with the incidence of 0.04–0.13% (1). GA is more commonly observed in female (ratio 3: 1) (2). From the embryogenic aspect, the disease is caused by gallbladder and cystic duct failure to bud off from the common bile duct during the first trimester of gestation (3). GA usually is asymptomatic and diagnosis is made incidentally during autopsy. In some patients, GA imitates signs and symptoms of biliary colic, cholelithiasis or cholecystitis with cystic duct obstruction or as sclero-atrophic gallbladder (4).According to our knowledge, the rare presentation of GA is recurrent cholangitis. Here in this paper, we wish to present a case of gallbladder agenesis and choledochogastric fistula who had recurrent cholangitis.

## Case Presentation

A 70- year- old man presented to Emergency Department of Modarres Hospital with 2 days history of abdominal pain, nausea, and vomiting. The patient described the pain in right upper quadrant, colic in nature, dull and exacerbated with meal. He was icteric. The patient had no significant past medical history except diabetes mellitus, he had no previous operation, and had no known drug allergies. Vital sign revealed blood pressure 90/60 mmHg, body temperature 38.5 ºCelsius degree and heart rate 110/min. Physical examination demonstrated tenderness in RUQ and Murphy’s sign was positive.

**Figure 1:**
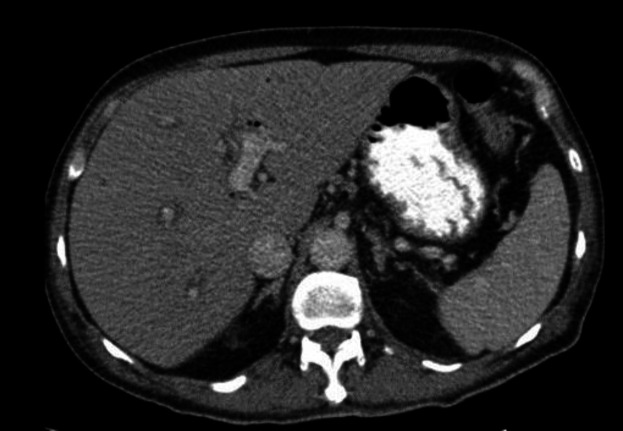
The CT scan showed evidence of pneumobilia in biliary ducts

Vital sign revealed blood pressure 90/60 mmHg, body temperature 38.5 ºCelsius degree and heart rate 110/min. Physical examination demonstrated tenderness in RUQ and Murphy’s sign was positive. The laboratory findings have shown in table 1. With the possibility of cholangitis, he was admitted and initial management include bowel rest, IV fluid hydration and IV antibiotics were started. An abdominal ultrasound (US) demonstrated contracted gallbladder with extrahepatic and intrahepatic dilated ducts and a dilated common bile duct (10mm). The CT scan was done and showed evidence of pneumobilia in biliary ducts (figure 1). He underwent emergent endoscopic retrograde cholangiopancreatograph (ERCP) and sten placement without sphincterotomy due to low platelet count and high INR level. In ECRP, papilla was edematous and one supra-papillary fistula (spontaneous) were seen. Cannulation of CBD via fistula was done. CBD was 10mm and contained multiple filling defects. Dilatation with balloon TTS8-10 was done. Copious amount of sludge and small stones were extracted with balloon. One plastic stent 8mm was inserted complete drainage was seen.

**Figure 2:**
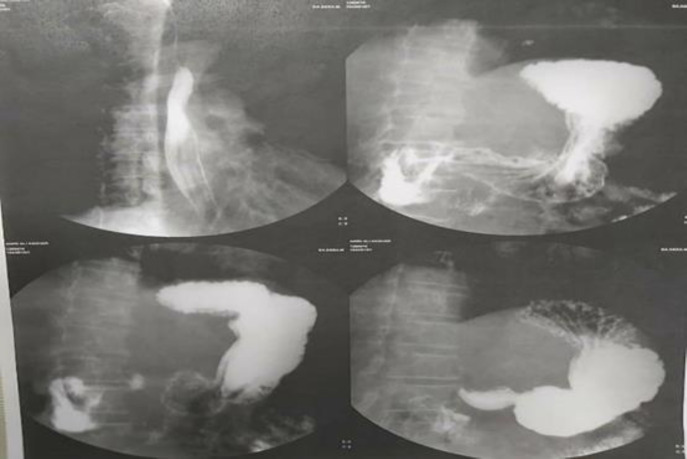
Upper GI series reported no evidence of fistula

**Figure 3:**
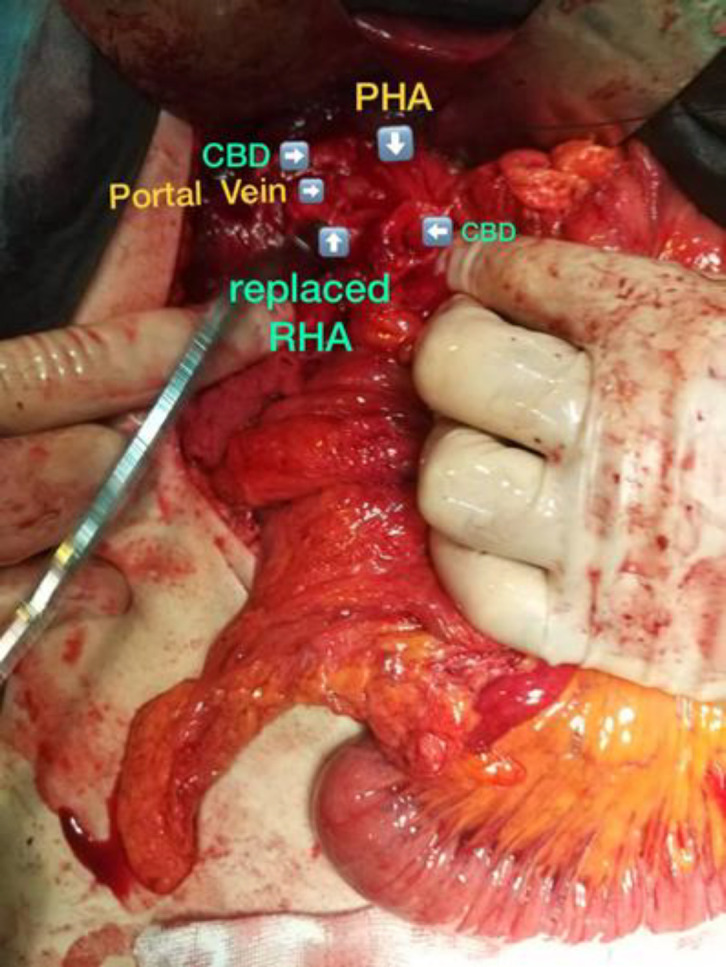
Had replaced right hepatic artery

According to report of ERCP and possibility of CBD to duodenum fistula, upper GI series was done and reported no evidence of fistula (figure 2) After conservative treatment and ERCP and stent placement, he was discharged from hospital. After discharge, he presented to hospital with recurrent fever, icter, weakness and weight loss. He underwent laparotomy. Gallbladder was not seen in anatomical location (GA). CBD had fistula to gastric antrum just lower than bifurcation level of common hepatic duct that made it impossible to repair CBD after dissection. Furthermore, he had replaced right hepatic artery (figure 3) Therefore we decided to do Roux-en-Y hepaticojejunostomy.

**Table 1:** Primary lab test of patient

**Lab test**	
**WBC**	4400
**Hb**	12.8
**Plt**	76000
**ALT**	98
**AST**	76
**ALP**	517
**Bili .T**	7.44
**Bili. D**	4.8
**Urea**	48
**Cr**	1.23
**Na**	142
**K**	3.5
**INR**	2

WBC; White blood cells, Hb; Hemoglobin, Plt; Platelets, ALT; Alanine transaminase, AST; Aspartate transferase, ALP; Alkaline phosphatase, Bili .T; Bilirubin test, Bili. D; Bilirubin direct, Cr; Creatinine, Na; Sodium test, k; Potassium test, INR; International normalised ratio

## Discussion

We reported a case with GA and CBD fistula to gastric antrum who presented to hospital with cholangitis and recurrent fever and weight loss. Finally, he underwent Roux-en-Y hepaticojejunostomy.

The gallbladder is derived from the foregut at fourth weeks of gestation. During the seventh week of gestation, the gallbladder and cystic duct develop a lumen followed by vacuolation of the hyperplastic epithelium. Any abnormality in this process may lead to agenesis of the gallbladder (5). There is some evidence showed that GA is related to some other congenital conditions such as cardiovascular abnormalities, atresia of the extra-hepatic biliary tract, intestinal malrotation, imperforate anus and some others (6). The differential diagnosis of GA are diverticulum of common hepatic duct and cyst of hepatic duct. Reviewing the literature demonstrated that preoperative investigation such as magnetic resonance cholangiopancreatography (MRCP) or ERCP has a good result in detection rate for choledochal cyst, anomalous abnormal pancreaticobiliary junction, choledocholithiasis, and a cholangiocarcinomas (7). 

Gallbladder agenesis is classified into three different groups according to the clinical manifestation; first group is that which is asymptomatic who is incidentally identified (35%), second one are symptomatic (50%), and the last one are those patients with multiple fetal anomalies who expires in the perinatal period (15–16%) (8). Due to the nonspecific sign and symptoms in the patients who are symptomatic, it is difficult to diagnose the GA completely and correctly before the operation (9, 10). The most usual and common symptoms of GA is like the other biliary tract disorders include right upper abdominal pain (90%), nausea and vomiting (66%), intolerance to fatty food (37.5%), dyspepsia, bloating or icter (8). The symptoms may appear because of the biliary dyskinesia and dysfunction of the sphincter of Oddi (11). The mechanism of its symptoms may be due to biliary pathologies such as duct stones and dyskinesia of biliary or nonbiliary pathogenesis such as esophagitis and duodenitis (12). In our case, it seems that combination of fistula between CBD and gastric antrum and biliary pathogenesis caused his symptoms. Spontaneous biliary gastrointestinal fistulas include cholecystoduodenal, cholecystogastric, cholecystocolonic, choledochoduodenal and choledochogastric usually occurred after chronic cholelithiasis (13). A fistula between CBD and gastric antrum is extremely rare. According to our knowledge, this case is a rare case to be reported in the English literature. In 1985, a case of choledocogastric fistula associated with a large calculus situated in stomach was reported (14). We decided to repair fistula during operation and reconstruct the biliary tract by Roux-en-Y hepaticojejunostomy. 
